# Prognostic significance of calcium-related genes in lung adenocarcinoma and the role of *TNNC1* in macrophage polarization and erlotinib resistance

**DOI:** 10.3389/fimmu.2025.1509222

**Published:** 2025-05-13

**Authors:** Jian Feng, Zhe Chen, Gaoming Wang, Yu Yao, Xuewen Min, Jing Luo, Kai Xie

**Affiliations:** ^1^ Department of Thoracic Surgery, Shanghai Chest Hospital, Shanghai Jiao Tong University School of Medicine, Shanghai, China; ^2^ Department of Cardiothoracic Surgery, The Fourth Affiliated Hospital of Soochow University, Suzhou, China; ^3^ Department of Thoracic Surgery, Xuzhou Central Hospital, Clinical School of Xuzhou Medical University, Xuzhou, China; ^4^ Department of Respiratory Medicine, Nanjing Second Hospital, Nanjing University of Chinese Medicine, Nanjing, China; ^5^ Department of Jurong Hospital Affiliated to Jiangsu University, Zhenjiang, China; ^6^ Department of Cardiothoracic Surgery, Jinling Hospital, Medical School of Nanjing University, Nanjing, China

**Keywords:** lung adenocarcinoma, calcium-related genes, prognostic signature, TNNC1, erlotinib resistance, macrophage M2 polarization

## Abstract

**Background:**

Calcium signaling is critical in tumorigenesis. This study analyzed the characteristics of a calcium-related prognostic genes (CRPGs) signature in lung adenocarcinoma (LUAD) for prognostic value and explored *TNNC1* as a potential therapeutic target for erlotinib resistance.

**Methods:**

Clinical and RNA sequencing data from LUAD patients were obtained from the TCGA and GEO databases. CRPGs were identified through univariate Cox and Kaplan-Meier survival analyses. Calcium-related subtypes were determined via unsupervised clustering. A prognostic signature was constructed and validated using external datasets. Differences in immune infiltration and potential mechanisms in LUAD were explored using seven algorithms. The relationship between signature genes, chemotherapy sensitivity, and potential targeted therapies was evaluated. Potential drug targets were identified using Mendelian randomization (MR) and phenome-wide association studies (PheWAS). The association between *TNNC1*, erlotinib resistance, and macrophage M2 polarization was investigated through *in vitro* experiments.

**Results:**

The study identified 33 CRPGs and four subtypes among LUAD patients. The prognostic signature, comprising nine CRPGs, accurately predicted 1-, 2-, and 3-year overall survival. *TNNC1* was identified as a crucial tumor suppressor gene and potential drug target. Down-regulation of *TNNC1* decreased the IC_50_ value of erlotinib in LUAD cells and inhibited macrophage M2 polarization.

**Conclusion:**

This study developed a reliable prognostic signature based on nine CRPGs for predicting LUAD patient outcomes. *TNNC1* may enhance LUAD cell resistance to erlotinib through macrophage polarization to the M2 phenotype.

## 1 Introduction

Lung cancer (LC) is a leading cause of cancer mortality worldwide, with a five-year relative survival rate of less than 20% (1). Lung adenocarcinoma (LUAD) accounts for roughly 50% of LC cases, with incidence rates rising annually (2). Although advancements in LUAD treatments, such as targeted therapies and immunotherapy, have been made, only a small subset of patients benefit, and overall survival (OS) rates remain low. Early metastasis and late diagnosis significantly contribute to the high mortality associated with LUAD (3). Therefore, there is an urgent need to develop biomarkers for early-stage diagnosis and treatment of LUAD.

Calcium ions play a crucial role in tumor development, influencing processes such as cell proliferation, differentiation, and apoptosis (4). Intracellular calcium homeostasis is meticulously regulated by ion channels, ATPase pumps, and exchangers (5). Dysregulation in these channels or pumps is linked to carcinogenesis. For example, malfunctions in *STIM* and *ORAI1*-mediated calcium storage and transport signaling can impede physiological and pathophysiological activities, such as breast tumor cell migration and metastasis (6), as well as vascular smooth muscle cell proliferation and migration (7). The expression of calcium-related proteins varies among cancer subtypes; for instance, *TRPV6* overexpression is significantly associated with the triple-negative breast cancer subtype due to increased *TRPV6* gene copy numbers (8, 9). However, the prognostic significance of calcium-related gene expression in LUAD is underexplored.

Calcium signaling dysregulation has emerged as a widespread adaptation in various malignancies, with discrete alterations occurring at different stages of cancer progression (10). In prostate cancer, altered calcium homeostasis depends on changes in the ratio of influx/efflux and storage of calcium compared with non-tumoral cells, with alterations in plasma membrane and endoplasmic reticulum channels being primarily responsible for abnormal intracellular calcium levels (11). In breast cancer, calcium-dependent kinases such as *CaMKK2* are expressed in both cancer cells and stromal cells, contributing to tumor growth and immune-suppressive status in the tumor microenvironment (12). High extracellular calcium levels have been associated with more aggressive and invasive breast cancers and increase the risk of bone metastasis in both breast and prostate cancers. Of particular interest to our study is the troponin C family member *TNNC1*, a calcium-binding protein classically associated with muscle contraction regulation. Recent evidence suggests troponin family members exhibit abnormal expression in various tumors, with *TNNC1* specifically demonstrating tumor-suppressive properties in certain cancer types. The dysregulation of calcium homeostasis is now recognized as an emerging feature of cancer, playing essential roles in the initiation and progression of malignant diseases, with the endoplasmic reticulum functioning as a major intracellular calcium store that modulates calcium homeostasis in coordination with other organelles (13). Targeting calcium signaling mediators has become a promising strategy for developing novel anticancer therapies across multiple cancer types (12). However, the prognostic significance of calcium-related gene expression, particularly *TNNC1*, in LUAD remains underexplored.

Erlotinib, a first-generation epidermal growth factor receptor tyrosine kinase inhibitor (EGFR-TKI), has demonstrated efficacy in NSCLC patients with sensitizing mutations (14). However, most patients eventually develop acquired resistance, leading to treatment failure (15). Thus, exploring EGFR-TKI resistance mechanisms and identifying genes to overcome resistance in NSCLC is critical for therapeutic advancements.

In this study, we identified 33 calcium-related prognostic genes (CRPGs) and categorized them into four distinct clusters (C1, C2, C3, C4) based on unique prognostic, biological, and immunological characteristics. Using LASSO-Cox regression analysis, we refined these CRPGs and developed a calcium-related risk score prognostic signature, termed CRPGscore, comprising nine key CRPGs. Independent test and validation sets confirmed the accuracy and specificity of this signature, suggesting its potential as an independent prognostic factor for OS in LUAD. Stratified analyses revealed that the high-risk group had significantly shorter OS durations compared to the low-risk group (*P* < 0.05). Additionally, the CRPGscore inversely correlated with immune cell content. Using Connectivity Map (CMap) analysis, we identified potential compounds and chemotherapy agents that could target this signature and modulate its carcinogenic effects. MR and PheWAS analyses identified *TNNC1* as potential drug targets for LUAD. Furthermore, through eight machine learning models, we identified *TNNC1* as a key tumor suppressor gene in LUAD. *In vitro* experiments demonstrated *TNNC1* may enhance LUAD cell resistance to erlotinib through macrophage polarization to the M2 phenotype.

## 2 Materials and methods

### 2.1 Data collecting

The training set consisted of gene expression data (HTSeq-FPKM) and associated clinical information from the TCGA database, including 458 LUAD patients with complete follow-up data and a follow-up duration longer than 30 days. Patients lacking comprehensive survival information were excluded. The validation set followed identical inclusion criteria and incorporated datasets GSE68465 (439 LUAD patients) and GSE72094 (386 LUAD patients) from the GEO database. Data from the TCGA-LUAD, GSE68465, and GSE72094 datasets were harmonized using the “Combat” algorithm from the R package “sva” (16). We have supplemented detailed steps and parameter settings for integrating data from different platforms using the “Combat” algorithm to ensure transparency and reproducibility of the data integration process. Specifically, we have supplemented detailed steps and parameter settings for integrating data from different platforms using the “Combat” algorithm. Specifically, to eliminate batch effects between the TCGA-LUAD, GSE68465, and GSE72094 datasets, we employed the “Combat” algorithm from the R package “sva” for data integration. First, we performed log2 transformation on the gene expression matrices of the three datasets to obtain approximately normally distributed expression values. Subsequently, we identified and retained genes common to all three datasets to construct a merged expression matrix containing all samples. When using the ComBat function, we set the parameter par.prior=TRUE to enable the empirical Bayesian method for enhancing the robustness of batch effect correction, while setting mean.only=FALSE to adjust both location and scale parameters simultaneously. To preserve biologically significant variation, we incorporated important clinical covariates (such as age, gender, and tumor stage) using the mod parameter, ensuring that the influence of these variables was retained during the batch correction process. After batch correction, we verified the effective removal of batch effects using principal component analysis (PCA) and t-SNE visualization methods, confirming that samples no longer clustered according to their original datasets but rather distributed based on their biological characteristics.

To further investigate the mechanisms of erlotinib resistance (ER), we incorporated resistance data from four GEO datasets of LUAD cell lines:

GSE75308-PC9: This dataset contains gene expression profiles of PC9 cell line and its erlotinib-resistant derivatives, generated using the Illumina HumanHT-12 V4.0 expression microarray platform. It comprises 12 samples (6 sensitive and 6 resistant strains).

GSE67051-PC9: This dataset includes gene expression data of PC9 cell line at different time points of erlotinib exposure, using the Affymetrix Human Gene 1.0 ST array platform. It contains 15 samples, documenting the dynamic process of resistance acquisition.

GSE67051-HCC827: Expression data of HCC827 cell line and its resistant derivatives from the same study, using the identical Affymetrix platform, comprising 12 samples.

GSE123031-PC9: This dataset contains RNA-seq data of PC9 and various acquired resistant PC9 variant cell lines, generated using the Illumina HiSeq 2500 platform, comprising 9 samples.

These cell line datasets were downloaded from the GEO database and processed using the same standardization procedures as the primary clinical datasets. For microarray data, we employed the RMA algorithm for background correction and normalization; for RNA-seq data, we applied the same HTSeq-FPKM normalization method used for TCGA data. Prior to integrative analysis, all cell line datasets underwent batch effect correction using the Combat method as described above.

**Figure 1:**
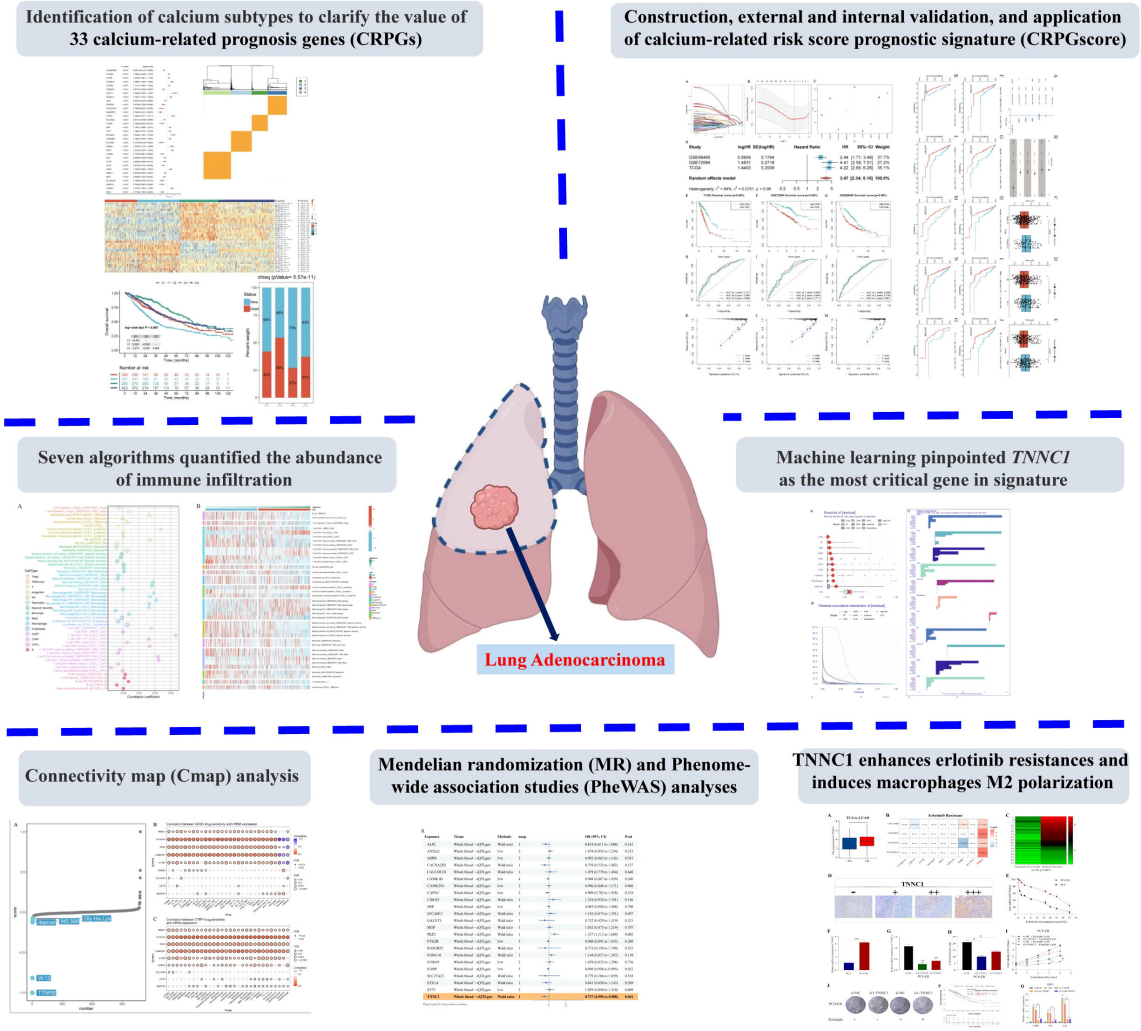
The flowchart of the overall study.

A comprehensive flowchart of the study is depicted in **Figure 1**.

### 2.2 Specimens and cell lines

Tissue samples were obtained from the Fourth Affiliated Hospital of Soochow University. Clinical data for these patients were retrieved from their medical records. This study was approved by the Ethics Committee of the same institution, and informed consent was obtained from all participants. Additionally, the study adhered to the ethical principles outlined in the Declaration of Helsinki, as published in the British Medical Journal (July 18, 1964).

The PC9 and THP-1 cell lines were acquired from the Shanghai Institutes for Biological Sciences (Shanghai, China). An erlotinib-resistant PC9 cell line (PC9-ER) was developed over six months by progressively increasing the erlotinib concentration from 0.1 μM to 40 μM using a stepwise incremental method. Both PC9 and PC9-ER cells were cultured in DMEM supplemented with 10% fetal bovine serum and antibiotics, maintained in a humidified atmosphere with 5% CO_2_ at 37°C. THP-1 cell line was maintained in RPMI 1640 medium supplemented with 10% heat-inactivated fetal bovine serum, 2 mM L-glutamine, and 1% penicillin/streptomycin in a humidified incubator at 37°C with 5% CO_2_.

### 2.3 Identification of calcium-related prognostic genes

Calcium-related genes were sourced from GeneCards, selecting those with a relevance score ≥ 8. Univariate Cox survival analysis and Kaplan-Meier (KM) survival analysis were performed on the TCGA-LUAD, GSE68465, and GSE72094 datasets using the “survival” package. A stringent *P*-value threshold of < 0.001 was applied to ensure result reliability, leading to the identification of 33 CRPGs.

### 2.4 Unsupervised clustering to identify CRPG subtypes and assess their value in LUAD

Unsupervised clustering was conducted using the “ConsensusClusterPlus” R software package to identify novel subtypes (17). Subtypes were analyzed using the “survival” and “survminer” packages, employing KM survival analysis and log-rank tests. Mesenchymal and immune cell presence in malignant tissues was estimated using the ESTIMATE algorithm, with tumor purity calculated for different molecular subpopulations (18). Immune cell abundance across molecular subpopulations was assessed using MCP-Counter (19). The GSVA algorithm was employed to investigate significant TME characteristic differences (20, 21). Additionally, HLA gene expression levels were compared between subtypes.

### 2.5 Construction, validation and application of the prognostic signature

LASSO-Cox regression analysis was utilized to refine the 33 CRPGs, eliminating collinearity with TCGA LUAD patients serving as the training set. This process led to the development of a calcium-related risk score prognostic signature, calculated by multiplying the β (Coef) values by the expression levels of CRPGs. The risk score formula used was: Risk score = (β1*CRPG1 + β2*CRPG2 + β3*CRPG3 +… + βn*CRPGn), where β signifies the CRPG coefficient (22, 23). The median risk score was used as a cut-off threshold to classify patients into high- and low-score groups. KM survival analyses were carried out to evaluate these groups further. The accuracy of the risk model was measured by calculating the area under the curve (AUC) for predicting 1-, 2-, and 3-year OS outcomes.

Validation sets GSE68465 and GSE72094 were similarly analyzed. Univariate and multivariate regressions, visualized through forest plots, as well as KM survival analyses, were conducted to verify the validity of the signatures. Stratified analyses of clinical variables, including age, gender, stage, and T and N classifications, were performed to compare OS between the high- and low-risk groups.

### 2.6 Immune landscape analysis related to the CRPGs-associated risk model

To assess immune infiltration in patients, seven algorithms were employed: MCPcounter (19), CIBERSORT (24), xCell (25), TIMER (26), EPIC (27), Cibersort-ABS (28), and QUANTISEQ (29). These algorithms were used to compare the high-risk and low-risk groups, identifying differences in immune infiltration. Pearson correlations determined the relationship between risk scores and immune cell content. Normalized enrichment scores (NES) were calculated using the Hallmark gene set with the ‘GSEA’ package to compute NES and false discovery rate (FDR). Gene set enrichment analysis (GSEA) and Kyoto Encyclopedia of Genes and Genomes (KEGG) analyses were performed on the Hallmark gene set for both high-risk and low-risk groups.

### 2.7 Connectivity map analysis

Drug sensitivity analysis was carried out using the GSCA database, based on gene expression data (30). GSCALite provided a collection of 750 small molecule drugs from GDSC and CTRP, using gene expression data to identify differentially expressed genes associated with these drugs in samples with various risks of drug response. Among these differential genes, the top 150 that were significantly up-regulated or down-regulated were selected as signature-related markers. The CMap_gene_signatures. RData file, obtained from the database website (https://www.pmgenomics.ca/bhklab/sites/default/files/downloads), containing 1,288 compound-related features, was used to calculate the matching score. The analysis methodology adhered to those described in previous publications (31, 32).

### 2.8 Machine learning methods for filtering feature model construction and validation

We applied the train function from the caret package to train multiple machine learning models and used the explain function from the DALEX package to interpret these models. The predict function was used to evaluate the accuracy of each model and generate ROC curves, while the variable_importance function from the DALEX package was employed to calculate the importance of variables within the models. Reverse cumulative residual distribution plots and residual boxplots were generated to assess model performance. The top 10 most important genes for each model (as shown in the variable importance ranking plot) were identified. The root mean square error (RMSE) was used as the loss function, indicating the degree to which excluding a variable impacts the predicted values of the response variable, where larger RMSE values imply greater variable importance.

### 2.9 Mendelian randomization analysis

MR analyses were conducted using the R package TwoSampleMR V0.5.6.24 (33). LUAD data, identified by code ieu-a-984, were sourced from the Open GWAS IEU website (https://gwas.mrcieu.ac.uk/) as the outcome, while cis-eQTL data from eQTLGen served as the exposure (34). Screening criteria for the exposure file included a p-value threshold of 5e-8, a correlation coefficient of 0.001, a distance of 10,000 kb, and a minor allele frequency greater than 0.01, focusing on cis-loci within 1 MB upstream and downstream of the gene center. After data loading, outcome harmonization was performed using built-in functions. MR estimates for each SNP were calculated using the Wald ratio method, while the inverse-variance weighted (IVW) method was applied when multiple SNPs were present to compute a weighted mean of the ratio estimates, with weights based on the inverse variance of the ratio estimates. Horizontal pleiotropy was assessed by evaluating whether the MR Egger intercept significantly differed from 0 when at least three SNPs were analyzed. Cochran’s Q tests assessed heterogeneity among Wald ratios (35). Significance was determined using FDR-corrected p-values, with an FDR threshold set at less than 0.05. Statistically significant findings in replication studies were recognized when nominal p-values were below 0.05.

### 2.10 Phenome-wide association analysis

To assess the horizontal pleiotropy of potential drug targets and possible side effects, we performed PheWAS using the AstraZeneca PheWAS Portal (https://azphewas.com/). Multiple testing corrections were applied, and a significance threshold of 1E-8 was established, as recommended by the AstraZeneca PheWAS Portal, to minimize the risk of false positives (36).

### 2.11 RNA extraction and quantitative real-time PCR

Experiments were executed following the manufacturer’s protocols. Total RNA from cells or tissues was extracted using the TRIzol reagent (Invitrogen, Carlsbad, CA). Reverse transcription was conducted with the PrimeScript RT kit (Takara, Cat: RR036A, KeyGEN) to synthesize cDNA, which was subsequently subjected to qRT-PCR analysis. Each qRT-PCR trial was performed in triplicate, and results were normalized to β-actin using the 2^-ΔΔCt^ method. The primer sequences utilized for qRT-PCR analysis were as follows: *ACTIN* forward, 5′-*GTCATTCCAAATATGAGATGCGT*-3′; *ACTIN* reverse, 5′-*GCATTACATAATTTACACGAAAGCA*-3′; *TNNC1* forward, 5′-*GTCTGACCTCTTCCGCATGT*-3′; *TNNC1* reverse, 5′-*ATGAGCTCCTCGATGTCGTC*-3′.

### 2.12 Western blot analysis

Total protein was extracted from the cells using RIPA lysis buffer containing protease inhibitors. Protein concentration was determined using a BCA Protein Assay Kit. Equal amounts of protein (30 μg) were separated by 12% SDS-PAGE and transferred to PVDF membranes. The membranes were blocked with 5% non-fat milk in TBST for 1 hour at room temperature and then incubated with primary antibodies against *TNNC1* (1:1000) and HA-tag (1:2000) overnight at 4°C. After washing with TBST, the membranes were incubated with HRP-conjugated secondary antibodies (1:5000) for 1 hour at room temperature. Protein bands were visualized using an enhanced chemiluminescence detection system. *GAPDH* served as a loading control.

### 2.13 siRNA construction and cell transfection

Small interfering RNA (siRNA) targeting *TNNC1* (RiboBio, Guangzhou, China) was transfected into PC9 or PC9-ER cells using Lipofectamine 3000 (Invitrogen, Carlsbad, CA, USA), following the manufacturer’s instructions. Forty-eight hours post-transfection, cells were harvested for qRT-PCR and other subsequent experiments. The siRNA sequences were as follows: si1-*TNNC1* sense sequence, 5′-*CGGUAGAGCAGCUGACAGA*-3′; si2-*TNNC1* antisense sequence, 5′-*AAGAUAAUGCUGCAGGCUA*-3′; si-NC sense sequence, 5′-*UAACGACGCGACGACGUAAtt*-3′; si-NC antisense sequence, 5′-*UUACGUCGUCGCGUCGUUAtt*-3′.

### 2.14 Construction of *TNNC1* overexpression system

The *TNNC1* overexpression system was constructed using lentiviral vectors. The full-length coding sequence of human *TNNC1* was amplified by PCR and cloned into a lentiviral expression vector with an HA-tag. The recombinant plasmid was verified by DNA sequencing. Empty vector was used as a control. Lentiviral particles were produced in HEK293T cells by co-transfection with packaging plasmids. PC9 cells were infected with lentivirus containing either *TNNC1*-HA or empty vector control, and stable cell lines were established by puromycin selection (2 μg/mL) for 2 weeks.

### 2.15 Colony formation assay

Cells were seeded at a density of 4 × 10^2^ cells per well in a 6-well plate. After overnight stabilization, cells were treated with erlotinib and incubated at 37°C until colonies formed and matured, with media changes every three days. After 10 days, colonies were fixed with 4% paraformaldehyde for 10 minutes, stained with crystal violet for 30 minutes, washed with PBS, and imaged.

### 2.16 CCK8 assay

PC9 and PC9-ER cells transfected with si-*TNNC1* or si-NC were seeded in 96-well plates at a density of 2 × 10^3^ cells per well and incubated for 6–8 hours to establish baseline values. They were then exposed to various concentrations of erlotinib for 72 hours. Following treatment, 10 μl of CCK-8 solution was added to each well and incubated for 2 hours. Cell viability was assessed by measuring absorbance at 450 nm. For M2 macrophage polarization, PMA-differentiated THP-1 cells (M0) were stimulated with 20 ng/ml IL-4 and 20 ng/ml IL-13 for 48 hours.

### 2.17 Macrophage differentiation

THP-1 cells were differentiated into macrophages using phorbol 12-myristate 13-acetate (PMA). The differentiation of THP-1 monocytes into macrophage-like cells was induced by PMA. Briefly, THP-1 cells were seeded at a density of 5 × 10^5^ cells/ml in complete medium containing 50 ng/ml PMA for 24 hours to induce initial differentiation into M0 macrophages. After PMA treatment, the medium was replaced with fresh complete medium without PMA, and cells were rested for an additional 24 hours. For M2 macrophage polarization, PMA-differentiated THP-1 cells (M0) were stimulated with 20 ng/ml IL-4 and 20 ng/ml IL-13 for 48 hours.

### 2.18 Macrophage polarization assessment

Differentiated cells were washed twice with PBS and then incubated with PBS containing 2 mM EDTA at 37°C for 10 minutes to detach cells from culture plates. After collection, cells were centrifuged at 300 × g for 5 minutes, and the supernatant was discarded. Cells were resuspended in 100 μl of flow cytometry buffer (PBS containing 0.5% BSA and 2 mM EDTA). Fc receptor blocking reagent was added and incubated at 4°C for 15 minutes to reduce non-specific binding. Fluorochrome-conjugated antibodies were then added: anti-human CD206-PE (1:50 dilution) and anti-human CD163-APC (1:50 dilution), and incubated at 4°C in the dark for 30 minutes. Following incubation, cells were washed twice with flow cytometry buffer and resuspended in 300 μl of flow cytometry buffer. Cell analysis was performed using a flow cytometer (BD FACSCalibur), with a minimum of 10,000 events collected per sample. Data were analyzed using FlowJo software (Tree Star Inc.), and M2 macrophage polarization was assessed by measuring the percentage of CD206 and CD163 positive cells and their mean fluorescence intensity (MFI). Appropriate isotype control antibodies were used as negative controls.

### 2.19 Statistical analysis

Data analysis was conducted using R4.1 software. T-tests were employed for normally distributed data, while the Wilcoxon rank-sum test was used for non-normally distributed data. The Kruskal-Wallis test and one-way analysis of variance (ANOVA) served as non-parametric and parametric methods, respectively, for intergroup comparisons. Univariate Cox survival analysis and Kaplan-Meier survival analysis were conducted using the “survival” package. Data analysis and graphical representation were facilitated by GraphPad Prism 9. All experiments were independently repeated at least three times. A statistical significance level of *P* < 0.05 was considered.

## 3 Results

### 3.1 Identification of calcium-related prognosis genes in LUAD

A comprehensive flowchart of the study is depicted in **Figure 1**.

**Figure 2:**
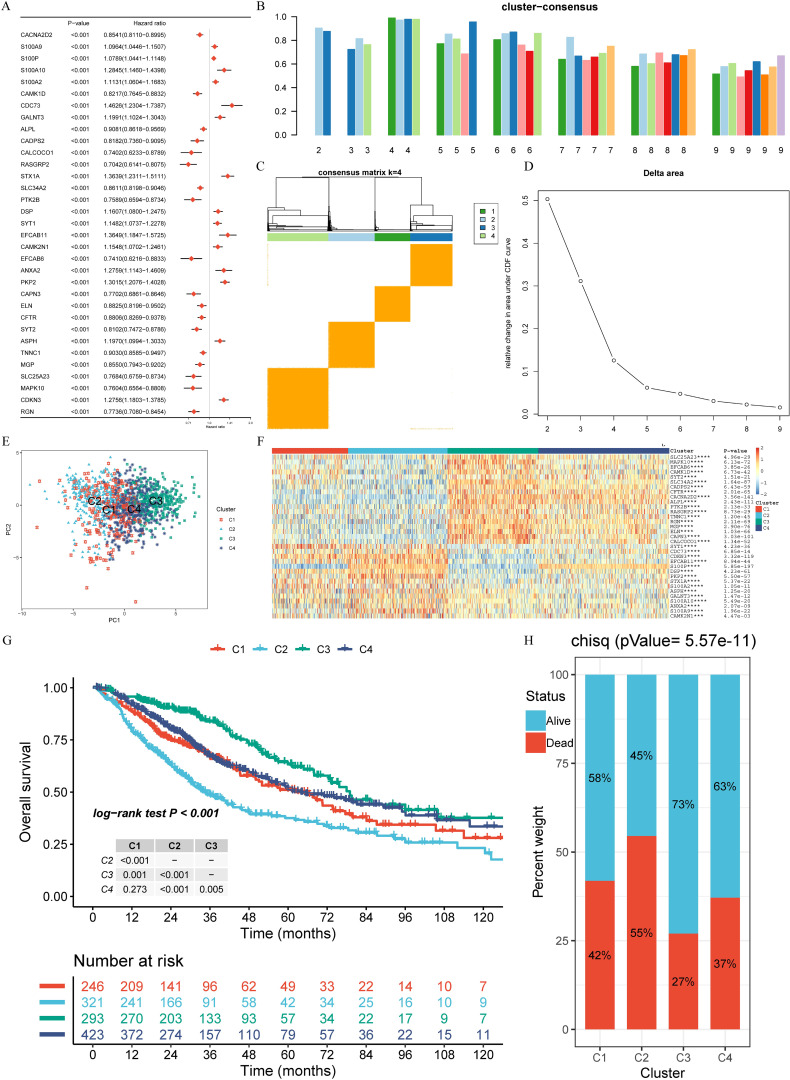
Calcium-related prognosis genes (CRPGs) expression profiling identifies four LUAD subtypes with distinct prognoses. **(A)** Univariate Cox survival analysis and Kaplan-Meier survival analysis for 33 calcium-related prognosis genes (CRPGs) in the TCGA-LUAD, GSE68465, and GSE72094 cohorts are shown in the forest map. **(B)** Assessment of average consistency within clusters. **(C)** Consensus clustering matrix for k = 4. **(D)** The cumulative distribution function (CDF) curve of the sample and delta area curve, reflecting the degree of variance of the area under the CDF curve for each number of categories k relative to k - 1. The horizontal axis represents the number of categories k and the vertical axis represents the relative change in area under the CDF curve. **(E)** Principal component analysis (PCA) revealed significant differences in transcriptome between the four clusters. **(F)** A heatmap displaying the expression of 33 CRPGs in different clusters. **(G)** The Kaplan–Meier curves show the overall survival for four clusters of LUAD patients (log-rank test). **(H)** Survival and mortality of each cluster (Chi-Squared test, *P* = 5.57E-11). ***P < 0.001, and ****P < 0.0001.

To ensure high sample quality, a rigorous screening process was implemented, selecting only samples with complete survival data and a follow-up duration exceeding 30 days. The R package “sva” and the “Combat” algorithm were utilized to minimize non-biological variations across the three datasets, culminating in the inclusion of 1,283 patients (TCGA-LUAD=458, GSE68465 = 439, GSE72094 = 386). Univariate Cox survival analysis (*P* < 0.001) and KM survival analysis (*P* < 0.001) conducted on 392 calcium-related genes with a relevance score of ≥ 8 led to the identification of 33 CRPGs (**Figure 2A**; **Supplementary Table S1**).

### 3.2 Evaluation of prognosis, immune infiltration, and biological function of CRPG clusters

Unsupervised clustering was applied to 1,283 LUAD tissues to identify novel subtypes based on the expression profiles of 33 CRPGs. Through rigorous analysis using cluster consensus, a consensus matrix, and a delta area plot, four clusters (C1, C2, C3, C4) were identified (**Figures 2B-D**; **Supplementary Table S2**). Principal component analysis (PCA) indicated significant transcriptional variations of CRPGs across these clusters (**Figure 2E**). All 33 CRPGs showed markedly different expression levels among the clusters (*P* < 0.0001) (**Figure 2F**). KM survival analyses and log-rank tests revealed substantial survival differences between most cluster pairs (*P* < 0.005, **Figure 2G**). The Chi-Squared test demonstrated diverse survival rates among clusters, with C3 exhibiting the highest survival rate at 73%, followed by C1 and C4 (58% and 63%, respectively), while C2 had the lowest survival rate at 45% (*P* = 5.57E-11, **Figure 2H**).

**Figure 3:**
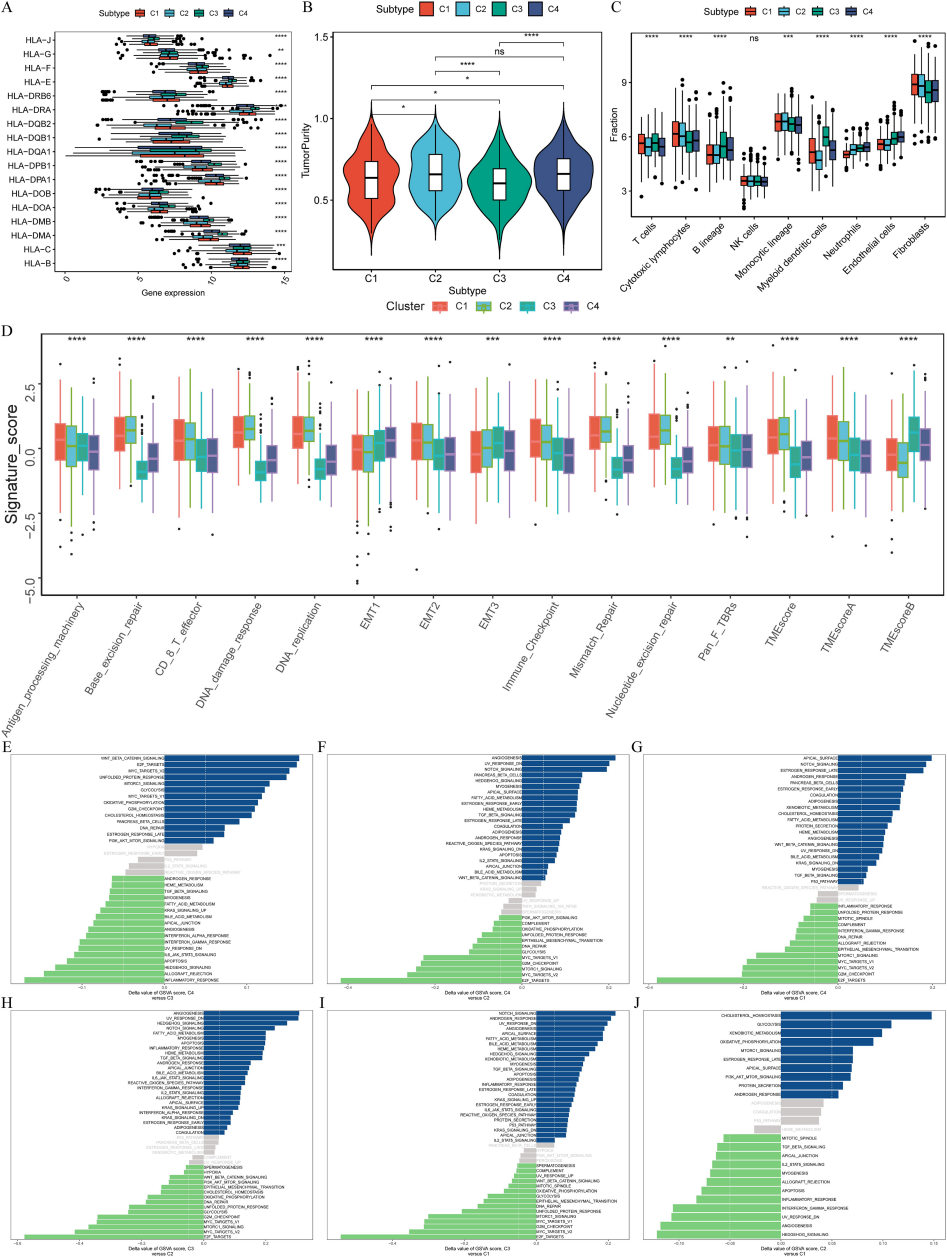
Distinct immunological features and pathways in four clusters. **(A)** Comparison of the expression levels of HLA genes among four clusters (ANOVA test, *P* < 0.01). **(B)** Intratumor heterogeneity (ITH) scores among four clusters (one-tailed Mann–Whitney U test). **(C)** Abundance of immune cell subpopulations (estimated by MCP-counter). **(D)** Contrast TME signature between the clusters C1, C2, C3 and C4 based on the GSVA algorithm. **(E–J)** Differences in pathway activities scored by GSVA between different CRPG clusters. The blue represented activated pathways and green represented inhibited pathways. *P < 0.05, **P < 0.01, ***P < 0.001, and ****P < 0.0001.

Further analysis of the subtypes revealed that C3 had the highest HLA gene expression and lowest tumor purity, indicative of stronger immune responses, whereas C2 displayed the highest tumor purity and the lowest immune cell infiltration, correlating with its poor prognosis (**Figures 3A-D**). GSVA and limma differential analysis uncovered significant pathway variations among the subtypes: C3 was enriched in immune response and T cell activation pathways, while C2 was characterized by enrichment in cell cycle and DNA replication pathways (**Figures 3E-J**). These differences in molecular characteristics likely explain the observed prognostic disparities and provide insight into potential precision treatment strategies for each subtype.

These findings emphasize the role of the 33 CRPGs in differentiating patients into four biologically distinct clusters, underscoring their potential prognostic and therapeutic significance. The molecular characteristic differences revealed by subtype analysis provided the basis for our prognostic model construction. Next, we developed a prognostic scoring model based on these 33 CRPGs and validated its predictive value in multiple independent cohorts.

### 3.3 Construction and external validation of calcium-related risk score prognostic signature

**Figure 4:**
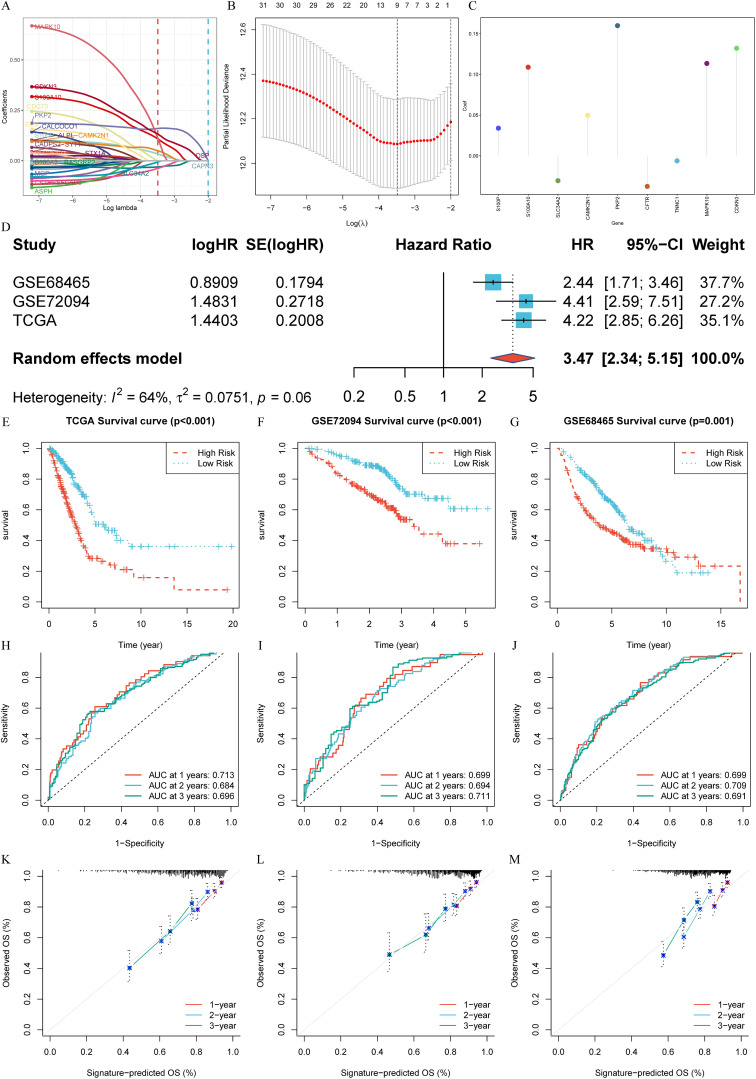
Construction and External validation of calcium-related risk score prognostic signature. **(A)** LASSO co-efficient value of 33 calcium-related prognosis genes (CRPGs). **(B)** 10-fold cross-validation for tuning parameter selection in the LASSO model. **(C)** The risk score for predicting the survival and prognosis of patients with LUAD. **(D)** A meta-analysis of the prognostic value of the immune signature model when used to predict outcomes in the TCGA-LUAD and GEO cohorts. **(E-G)**. Kaplan–Meier analyses demonstrate the prognostic significance of prognostic signature in **(E)** the TCGA-LUAD (*P* < 0.001), **(F)** the GSE72094 (*P* < 0.001), **(G)** the GSE68465 (*P* = 0.001). **(H-J)**. Time-dependent receiver operating characteristics (ROC) of **(H)** the TCGA-LUAD, **(I)** GSE72094 and **(J)** GSE68465. **(K-M)**. Calibration curves for risk score model in **(K)** the TCGA-LUAD, **(L)** GSE72094 and **(M)** GSE68465.

Utilizing the TCGA-LUAD dataset as the training set, we applied LASSO-Cox regression analysis to streamline the list of 33 CRPGs, removing collinearity and optimizing prognostic characteristics (**Figures 4A, B**). The process led to the development of a risk score prognostic signature, calculated by multiplying the β (Coef) values by the CRPG expression levels, resulting in the formula: CRPGscore = (0.034 × *S100P* expression) + (0.109 × *S100A10* expression) + (-0.031 × *SLC34A2* expression) + (0.050 × *CAMK2N1* expression) + (0.160 × *PKP2* expression) + (-0.038 × *CFTR* expression) + (-0.006 × *TNNC1* expression) + (0.113 × *MAPK10* expression) + (0.132 × *CDKN3* expression) (**Figure 4C**; **Supplementary Table S3**).

The biological roles of each CRPG within the signature further underscore their importance in shaping the prognostic model. For instance, *S100P* and *S100A10*, both calcium-binding proteins, are involved in regulating cell proliferation, differentiation, and migration. *S100P* is overexpressed in multiple cancers and linked to poor prognosis in LUAD, while *S100A10* contributes to membrane repair and drug resistance in tumor progression. Contrasting with these findings, S*LC34A2*, a sodium-phosphate cotransporter downregulated in LUAD, functions as a tumor suppressor gene by maintaining calcium-phosphate balance. Similarly, downregulated *TNNC1*, a novel finding in LUAD, is associated with extended survival, suggesting potential tumor-suppressive functions. On the other hand, overexpressed genes like *PKP2* and *CDKN3* may promote tumor progression by affecting calcium-dependent cell-cell adhesion and cell cycle regulation, respectively. The inclusion of *CAMK2N1* and *MAPK10*, involved in calcium-driven stress response and apoptosis, and CFTR, an immune microenvironment regulator, highlights the multifaceted roles of calcium signaling in LUAD progression. These insights provide a mechanistic basis for the prognostic utility of the CRPG signature.

To assess the predictive value of the signature, a random effects model was employed to conduct a prognostic meta-analysis across the GEO cohorts (GSE72094 and GSE68465) and the TCGA-LUAD cohort. This analysis indicated that the prognostic signature is a significant risk factor across datasets (HR: 3.47, 95% CI: 2.34-5.15, Weight = 100.0%). No significant heterogeneity was observed (I² = 64%, *t* = 0.0751, *P* = 0.06) (**Figure 4D**). Using the median risk score from the TCGA-LUAD scores as the cut-off value, patients across all three cohorts were classified into high- and low-score groups. The low-score group consistently showed significantly better OS compared to the high-score group in TCGA-LUAD (*P* < 0.001, **Figure 4E**), GSE72094 (*P* < 0.001, **Figure 4F**), and GSE68465 (*P* = 0.001, **Figure 4G**). ROC analysis was performed to evaluate the sensitivity and specificity of the signature for prognosis, using the AUC as the performance metric. The AUC values for the TCGA-LUAD training set at 1, 2, and 3 years were 0.713, 0.684, and 0.696, respectively (**Figure 4H**). For the GSE72094 test set, the AUC values were 0.699, 0.694, and 0.711, whereas the GSE68465 test set yielded AUC values of 0.699, 0.709, and 0.691 (**Figures 4I, J**). Moreover, the reliability of the ROC in predicting outcomes in LUAD patients was confirmed (**Figures 4K-M**).

These results highlight the robustness and reliability of the constructed prognostic signature in accurately predicting the prognosis of LUAD across multiple datasets.

### 3.4 Internal validation of CRPGscore prognostic signature

**Figure 5:**
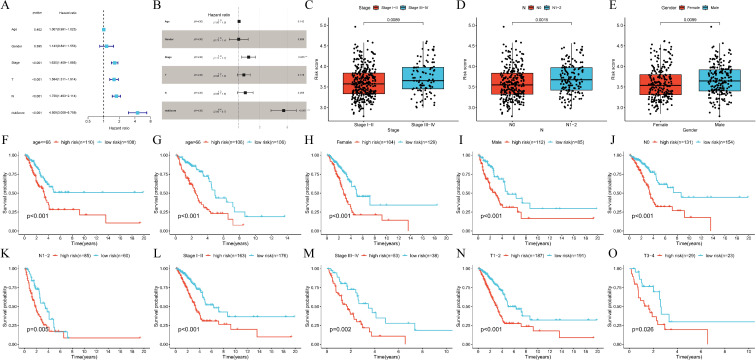
Internal validation of calcium-related risk score prognostic signature. **(A, B)**. **(A)** Univariate Cox regression and **(B)** multivariable Cox regression analysis of correlations between the risk score for over survival (OS) and clinical variables in TCGA-LUAD cohort. **(C-E)**. The relationships between the risk score and clinicopathological variables. **(C)** Stage (stage I-II vs. stage III-IV, *P* = 0.0089). **(D)** N stage (N0 vs. N1-2, *P* = 0.0015). **(E)** Gender (Female vs. Male *P* = 0.0099). N, lymph node metastasis. **(F, G)**. Subgroup analysis of Kaplan-Meier curves in different ages ≤66 (*P* < 0.001) and >66 (*P* = 0.005). **(H, I)**. Subgroup analysis of Kaplan-Meier curves in female (*P* < 0.001) and male (*P* < 0.001). **(J, K)**. Subgroup analysis of Kaplan–Meier curves in N0 (*P* < 0.001) and N1-2 (*P* = 0.005). N, lymph node metastasis. **(L, M)**. Subgroup analysis of Kaplan–Meier curves in different stage I-II (*P* < 0.001) and III-IV (*P* = 0.002). **(N, O)**. Subgroup analysis of Kaplan–Meier curves in T1-2 (*P* < 0.001) and T3-4 (*P* = 0.026). T, tumor size.

Based on the aforementioned analysis of the expression pattern and functional characterization of CRPGs in LUAD, we further evaluated the value of the constructed prognostic features for application in different clinical subgroups. Cox regression analysis was performed to evaluate the signature as an independent predictor in LUAD patients. The univariate Cox regression analysis showed that OS in LUAD was strongly associated with stage, T stage, N stage, and risk score (**Figure 5A**). Multivariate Cox regression analysis further confirmed that the risk score was a significant independent prognostic factor for LUAD patients (*P* < 0.001) (**Figure 5B**). An examination of the relationship between the signature and clinicopathological variables revealed that the CRPGscore was significantly higher in patients with stage III-IV compared to stage I-II (*P* = 0.0089, **Figure 5C**), higher in N1–2 compared to N0 (*P* = 0.0015, **Figure 5D**), and higher in males compared to females (*P* = 0.0099, **Figure 5E**). These findings suggest that a higher CRPGscore is linked with increased malignancy in LUAD. Stratification of LUAD patients based on clinicopathological variables was conducted to investigate the prognostic value for OS. Stratification analyses were performed based on age (*P* < 0.001; *P* < 0.001; **Figures 5F, G**), sex (*P* < 0.001; *P* < 0.001; **Figures 5H, I**), N stage (*P* < 0.001; *P* = 0.005; **Figures 5J, K**), stage (*P* < 0.001; *P* = 0.002; **Figures 5L, M**), and T stage (*P* < 0.001; *P* = 0.026; **Figures 5N, O**). Consistently across all stratified analyses, the high-score group exhibited significantly shorter OS than the low-score group, indicating that the signature’s prognostic value was not confounded by conventional clinical factors in LUAD patients.

### 3.5 Integrated analysis of the immune landscape, molecular pathways, and therapeutic relevance in LUAD

**Figure 6:**
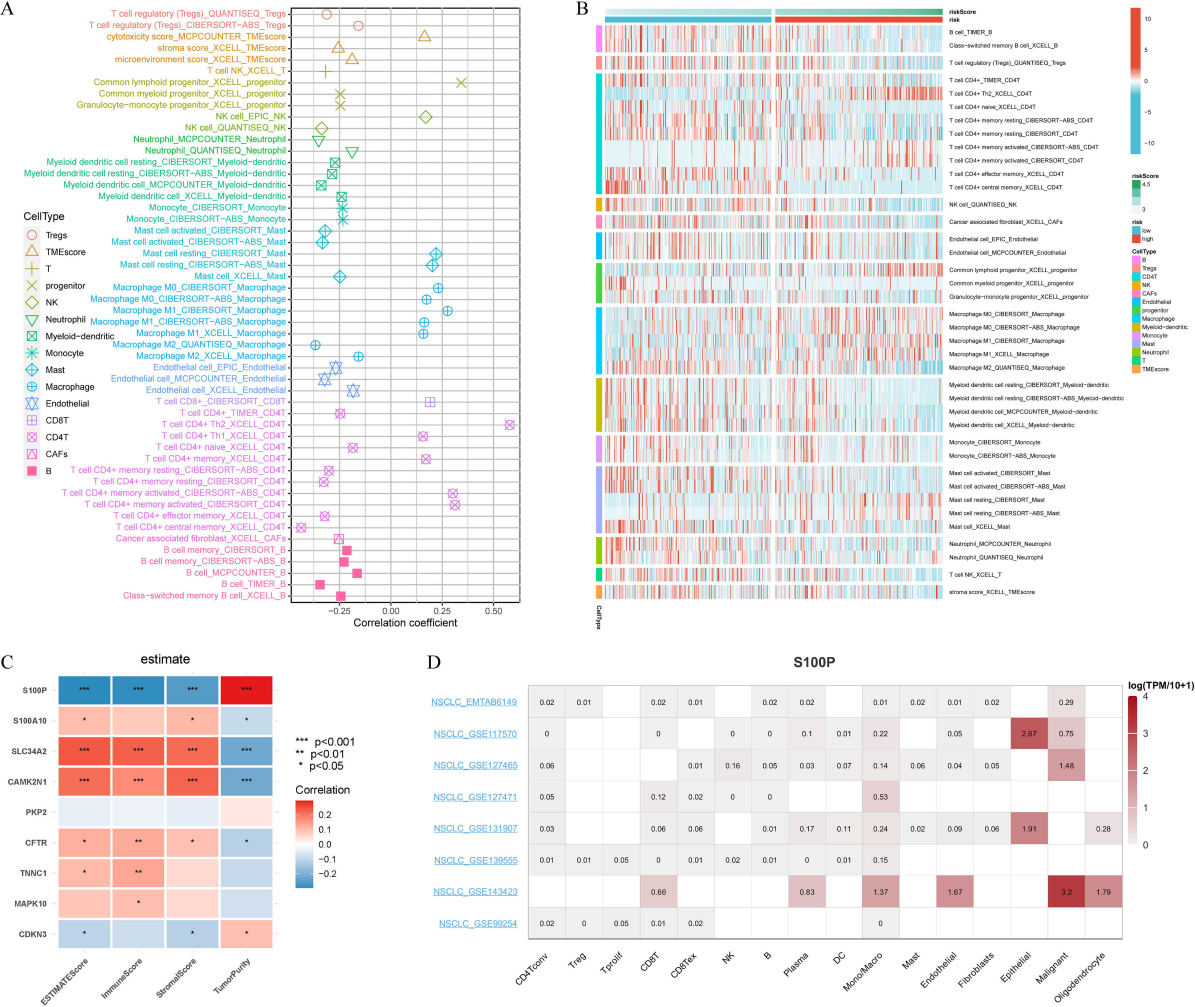
Immune cell landscape associated with signature in LUAD patients. **(A)** Seven algorithms (MCPcounter, CIBERSORT, xCell, TIMER, EPIC, Cibersort-ABS, QUANTISEQ) were used to compare the differences in immune cells infiltration (B cells, Tregs cells, CD4T+ cells, NK cells, CAFs cells, Endothelial cells, Progenitor cells, Macrophage cells, Myeloid-dendritic cells, Monocyte cells, Mast cells, Neutrophil cells, T cells, TMEscore) between the high and low riskscore groups. **(B)** Correlation of riskscore with immune cell infiltration evaluated using seven algorithms. **(C)** The estimate algorithm evaluated the correlation of 9 CRPGs with 4 scores (ESTIMATEScore, ImmunScore, StromalScore, and TumorPurity). **(D)** Single-cell expression analysis of S100P in NSCLC tissues. *P < 0.05, **P < 0.01, and ***P < 0.001.

We investigated the relationship between our gene signature and the tumor immune microenvironment in LUAD using seven algorithms (MCPcounter, CIBERSORT, xCell, TIMER, EPIC, CIBERSORT-ABS, and QUANTISEQ). The signature exhibited significant associations with various immune cell types, such as Tregs, T cells, NK cells, neutrophils, myeloid dendritic cells, monocytes, mast cells, and B cells, with notably higher infiltrating levels in the low-risk group than in the high-risk group (**Figures 6A, B**). Most CRPGs (except *PKP2*) were significantly correlated with immune-related scores, and *S100P* showed a strong positive relationship with TumorPurity (**Figure 6C**). Single-cell analyses further demonstrated enriched S100P expression in tumor-associated cells (**Figure 6D**).

Hallmark gene set-based GSEA and KEGG analyses revealed distinct pathway enrichments among high- and low-risk patients, indicating differing biological features (**Supplementary Figure S1**).

**Figure 7:**
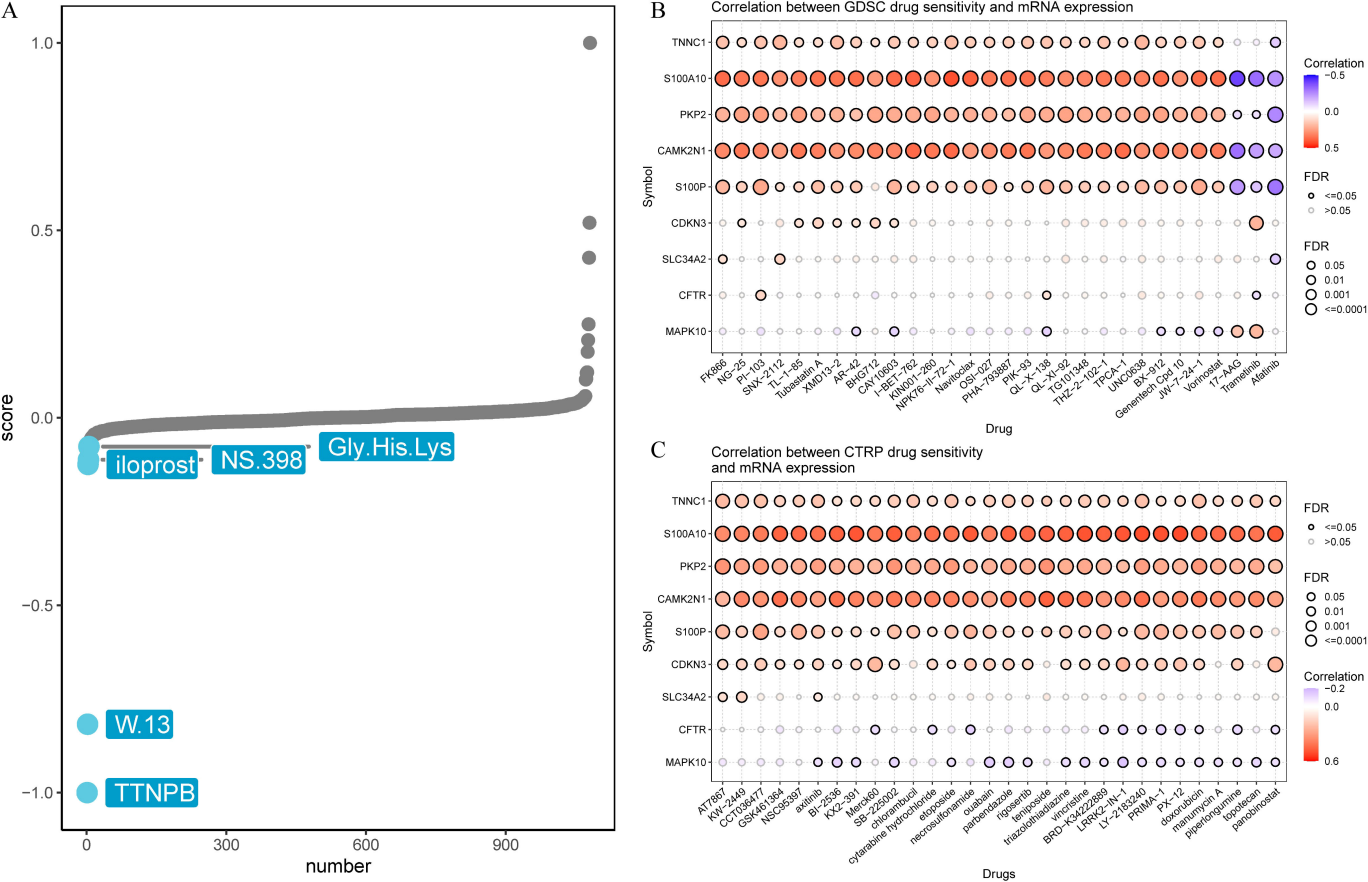
Prediction of potential drug targeting signature. **(A)** Candidate compounds may target signature based on the connectivity map (Cmap) analysis in LUAD. **(B)** Correlation between GDSC drug sensitivity and 9 CRPGs mRNA expression. **(C)** Correlation between CTRP drug sensitivity and 9 CRPGs mRNA expression. A negative correlation indicated that high gene expression made patients more sensitive to the drug, while a positive correlation indicated the opposite.

To explore the clinical relevance of the signature in anti-cancer therapy, we performed Connectivity Map (CMap) analysis using the top 150 differentially expressed genes in the high-risk group. Five candidate compounds (TTNPB, W.13, iloprost, NS.398, and Gly.His.Lys) were identified for their possible inhibitory effects on tumor-promoting mechanisms (**Figure 7A**). Notably, previous research has documented the inhibitory effects of NS.398 (37), TTNPB (38) and iloprost (39) on LUAD. Further integration of the GDSC and CTRP databases revealed that *TNNC1*, *S100A10*, *PKP2*, *CAMK2N1*, and *S100P* exhibited positive correlations with chemotherapy resistance (**Figures 7B, C**). Notably, *TNNC1*, *S100A10*, *PKP2*, *CAMK2N1*, *S100P*, and *CDKN3* in the CTRP dataset were linked to resistance across multiple drugs (**Figure 7C**). Collectively, these results underscore the potential clinical utility of these genes for predicting treatment outcomes.

### 3.6 Identification of *TNNC1* as a key suppressor gene in LUAD

**Figure 8:**
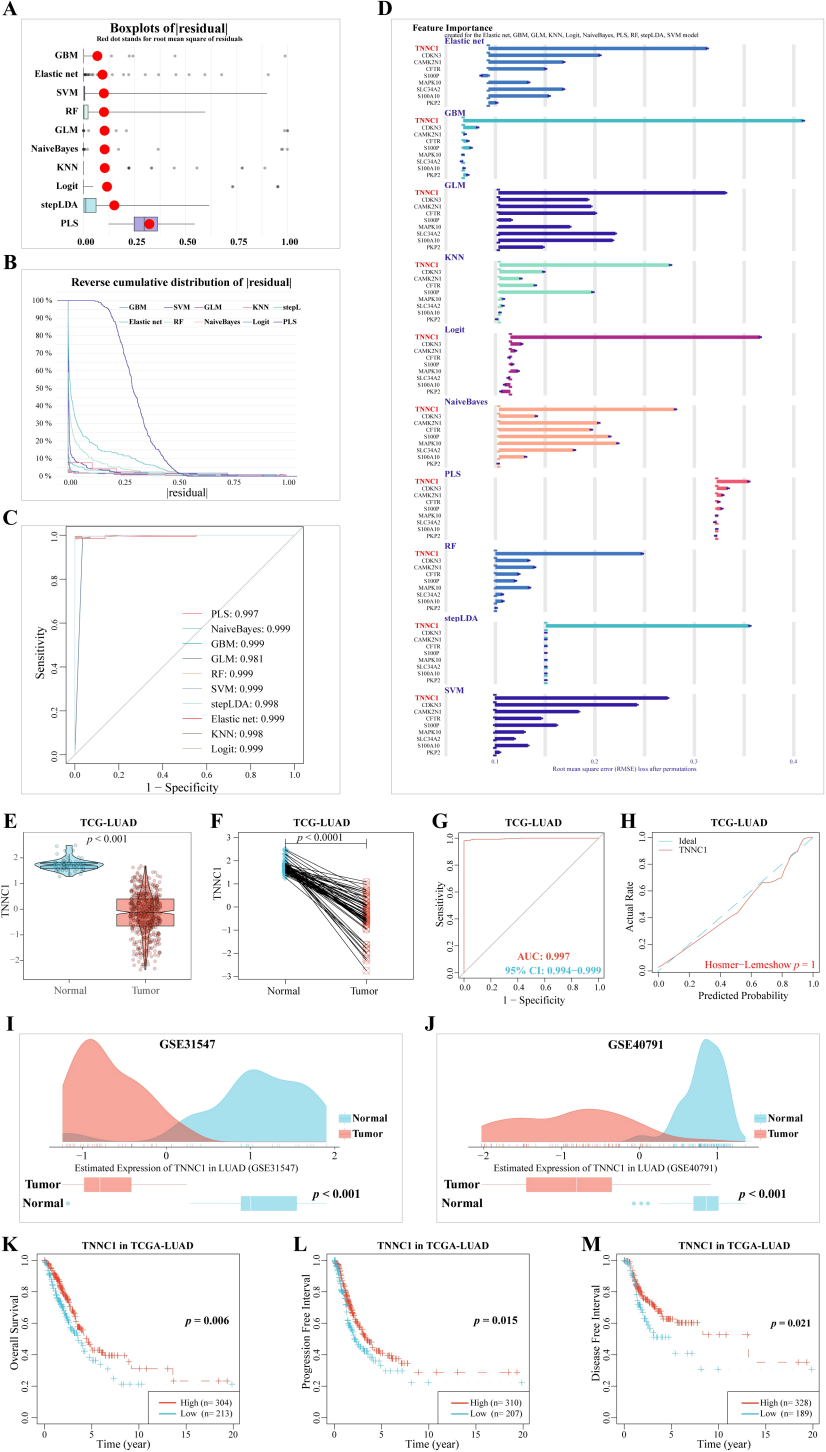
Machine learning methods for filtering feature model construction and validation. **(A)** Based on caret package, eleven algorithms (Logit, Gradient Boosting Machine (GBM), Support Vector Machine (SVM) learning, Linear Discriminant Analysis with Stepwise Feature Selection (stepLDA), Random Forest (RF) tree, K-Nearest Neighbor (KNM), Extreme Gradient Boosting (XGBoost), Multi-Step Adaptive MCP-Net (msaenet), Partial Least Squares (PLS), eXtreme Gradient Boosting (XGB), Generalized Linear model (GLM), and Naive Bayes classifier (NaiveBayes)) were used to construct boxplots of sample residuals. The X-axis value represents the quantile of outliers, and the red dot represents the mean. **(B)** Reverse cumulative distribution of residuals. The Y-axis value represents the percentile of the outlier. **(C)** ROC analysis of the eleven model (GBM, GLM, KNN, Logit, msaenet, NaiveBayes, PLS, RF, stepLDA, SVM, XGB model). **(D)** Feature Importance created for the GBM, GLM, KNN, Logit, msaenet, NaiveBayes, PLS, RF, stepLDA, SVM, XGB model. The X-axis value represents the root mean square error (RMSE) loss after permutations. **(E)** The mRNA expression of *TNNC1* in normal tissues and LUAD tissues. **(F)** The mRNA expression of *TNNC1* in paired normal tissues and LUAD tissues. **(G)** A ROC curve to test the value of *TNNC1* to identify LUAD tissues. **(H)** Hosmer-Lemeshow good of fit test. **(I)** In the GSE31547 dataset, *TNNC1* expression was higher in LUAD tumors (*P* < 0.001). **(J)** In the GSE40791 dataset, *TNNC1* expression was higher in LUAD tumors (*P* < 0.001). The **(K)** Overall Survival, **(L)** Progression Free Interval, **(M)** Disease Free Interval, KM survival curves of *TNNC1* in LUAD.

The TCGA-LUAD dataset was randomly divided into two subsets using the createDataPartition function from the caret package, designating one as the training set and the other as the test set. Diagnostic models were developed using nine CRPGs. Residual analysis demonstrated strong predictive capabilities for these models (**Figure 8A**), with minimal prediction errors evident in the inverse cumulative distribution of residuals across 11 methods (**Figure 8B**). ROC curves based on these residuals indicated excellent model performance (**Figure 8C**). Among the nine CRPGs, *TNNC1* consistently emerged as a critical tumor suppressor gene in LUAD across all algorithmic models (**Figure 8D**).

Building on this discovery, MR analysis employing 33 CRPGs further confirmed the tumor-suppressive role of *TNNC1*. The analysis revealed that increased *TNNC1* expression significantly reduces the risk of LUAD (OR = 0.737; 95% CI, 0.550–0.988; *P* = 0.041) (**Supplementary Figure S2A**). Additionally, a PheWAS analysis conducted via the Phenoscanner platform simulated potential drug-related side effects associated with TNNC1, and no significant adverse effects were observed (**Supplementary Figure S2B** and **Supplementary Table S4**). These results not only reinforce *TNNC1*’s protective role in LUAD but also highlight its potential as a novel therapeutic target.

Subsequent validation with the TCGA-LUAD dataset confirmed that *TNNC1* expression was significantly lower in LUAD tissues compared to adjacent normal lung tissues (**Figures 8E, F**, *P* < 0.001). ROC curves supported *TNNC1*’s robust differentiation between LUAD and normal lung tissues (AUC: 0.997, 95% CI: 0.994-0.999) (**Figure 8G**), and the Hosmer-Lemeshow test confirmed the model’s goodness of fit (*P* = 0.998, **Figure 8H**). Further validation using external datasets GSE31547 and GSE40791 affirmed that *TNNC1* expression is significantly lower in LUAD tissues compared to adjacent normal tissues (**Figures 8I, J**, *P* < 0.001). Kaplan-Meier survival analysis showed that patients with higher *TNNC1* expression had significantly better overall survival (*P* = 0.006, **Figure 8K**), progression-free interval (*P* = 0.015, **Figure 8L**), and disease-free interval (*P* = 0.021, **Figure 8M**) than those with lower expression. These findings highlight *TNNC*1’s downregulation in LUAD tissues and its close association with patient prognosis, suggesting its potential role as a prognostic biomarker.

### 3.7 *TNNC1* enhances the erlotinib resistances of LUAD cells and induces M2 polarization of macrophages

**Figure 9:**
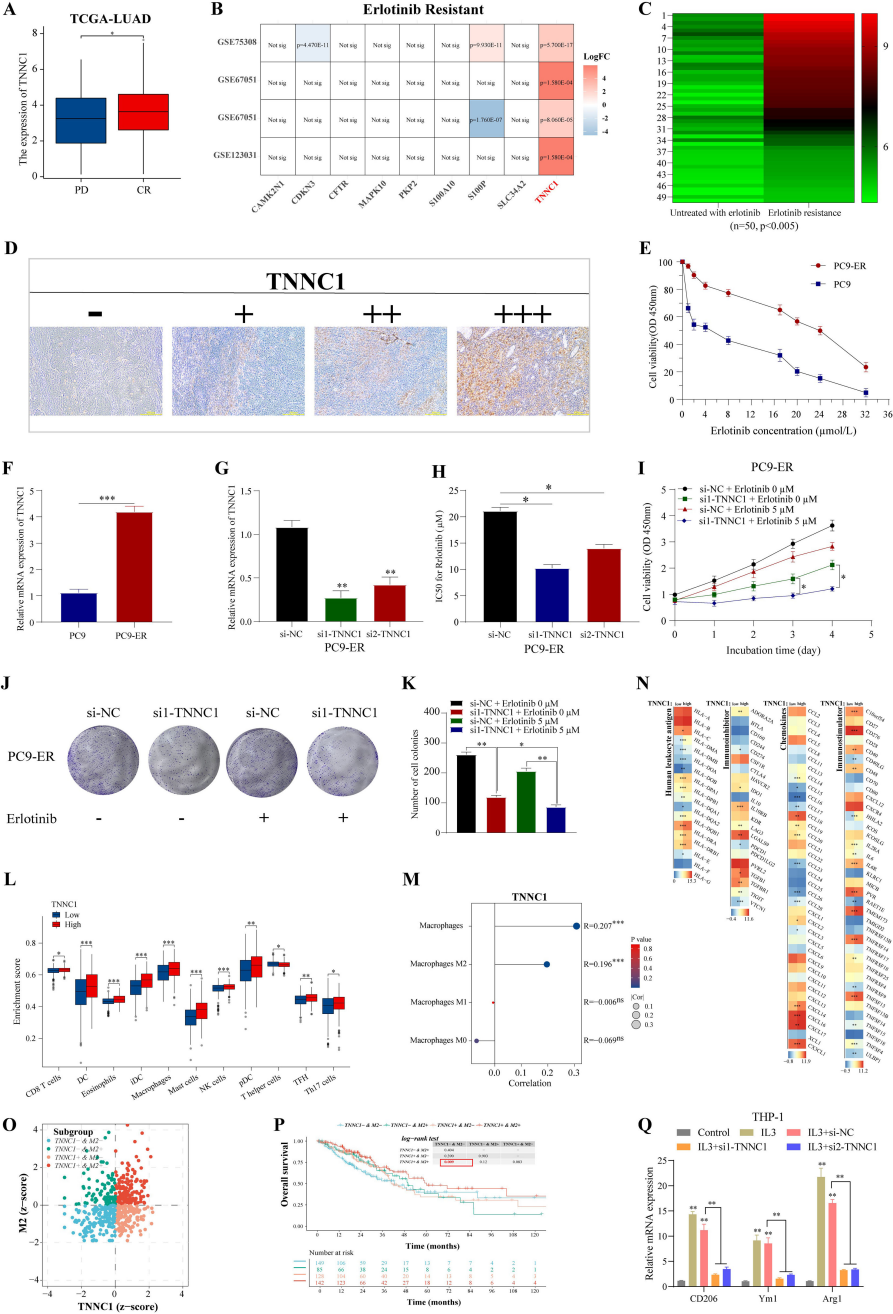
*TNNC1* enhances the erlotinib resistances of LUAD cells and induces M2 polarization of macrophages. **(A)** The mRNA expression of TNNC1 in erlotinib-treated LUAD patients with complete relief (CR) or clinical progressive disease (PD). **(B)** LUAD erlotinib-resistant cell dataset analyses expression of nine signature genes. **(C)** Heat map showed the mRNA expression of *TNNC1* in untreated with erlotinib group and erlotinib resistance group, ranging from green (low expression) to red (high expression). The column clustering generated by the IHC scores of the *TNNC1* staining. **(D)** Representative IHC images showing *TNNC1* expression (-, +, ++, +++) in LUAD tissues. Scale bar, 200 m. **(E)** IC_50_ values of gefitinib in PC9-ER and parental PC9 cells was examined by CCK8 assay. **(F)** The mRNA expression of *TNNC1* expression in PC9-ER and parental PC9 cells. **(G)** Validation of siRNAs-*TNNC1* knockdown efficiency in PC9-ER cells. **(H)** IC_50_ value of gemcitabine in PC9-ER cells transfected with siRNA targeting *TNNC1* (si*1/2-TNNC1*) by the cell counting kit-8 assay. **(I)** CCK8 assays were performed to determine the proliferation of siRNA-*TNNC1* PC9-ER cells treated with erlotinib. **(J, K)** Colony formation assays were used to evaluate the colony formation capacity of siRNA-*TNNC1* PC9-ER cells treated with erlotinib. **(L)** The expression of immunostimulatory gene, immunosuppressive gene, chemokine and human leukocyte antigen was different in high/low expression group of *TNNC1*. The left and right sides of the heat map showed low and high gene expression groups, respectively. The higher the average gene expression, the redder the color, and the lower the average gene expression, the bluer. **(M)** The infiltration of immune cells in TCGA-LUAD groups with high and low expression of *TNNC1* was analyzed by xCell. **(N)** The correlation between *TNNC1* expression and the proportion of total macrophages, M0, M1, M2 macrophages. **(O)** In the zcore scatter diagram of the sample, each scatter represents a sample, different colors represent different subgroups, and the horizontal/vertical coordinates correspond to the zcore of the two genes respectively. The definition of zcore ≤ 0 means low expression, zcore > 0 means high expression. **(P)** Kaplan-Meier survival analysis. **(Q)** After transfection with si-NC or si1/2-*TNNC1*, the expression levels of *CD206*, *Ym1* and *Arg1* in IL-3-treated THP1 cells were detected by RT-qPCR. THP1 cells not treated with IL3 were used as control. ER, erlotinib resistance. *P < 0.05, **P < 0.01, and ***P < 0.001.

Resistance to EGFR-TKIs is a common challenge in treating late-stage LUAD, often leading to relapse (40). Understanding the mechanisms behind this resistance is crucial for improving chemotherapy outcomes. TCGA-LUAD patient data revealed that *TNNC1* expression was significantly higher in patients classified in the progressive disease (PD) category compared to those with a complete response (CR) to EGFR-TKI treatment (**Figure 9A**). Further, an examination of erlotinib resistance (ER) datasets from LUAD cell lines (GSE75308-PC9, GSE67051-PC9, GSE67051-HCC827, GSE123031-PC9) consistently showed an increase in *TNNC1* expression in erlotinib-resistant LUAD cells compared to sensitive ones (**Figure 9B**). Elevated *TNNC1* expression in the ER group, as opposed to patients untreated with erlotinib, underscores its potential role as a key gene related to erlotinib resistance (**Figures 9C, D**). To explore *TNNC1*’s impact on erlotinib resistance, a PC9-ER cell model was developed using a dose-escalation approach with the PC9 cell line. The half-maximal inhibitory concentration (IC50) of erlotinib required for a 50% reduction in cell viability was assessed using the CCK-8 assay and a dose-response curve (**Figure 9E**). *TNNC1* mRNA levels were found to be higher in PC9-ER cells compared to PC9 cells (**Figure 9F**). To investigate further, PC9-ER cells were transfected with *TNNC1*-specific siRNA (si1/2-*TNNC1*) to knock down *TNNC1* expression, and transfection efficiency was confirmed 48 hours post-transfection via RT-qPCR (**Figure 9G**). Following *TNNC1* knockdown, the IC50 value of erlotinib in PC9-ER cells decreased compared with the control (**Figure 9H**). The CCK-8 assay results showed that downregulation of *TNNC1* significantly reduced the IC_50_ value of erlotinib in these cells (**Figure 9I**). In addition, *TNNC1* downregulation markedly inhibited PC9-ER cell proliferation as indicated by CCK-8 proliferation and colony formation assays (**Figures 9J, K**). To establish a direct causal relationship between *TNNC1* overexpression and erlotinib resistance, we overexpressed *TNNC1* in the erlotinib-sensitive PC9 cell line. Western blot confirmed successful expression of HA-tagged *TNNC1* in PC9 cells (**Supplementary Figure S3A**). Cell viability assays demonstrated that *TNNC1*-overexpressing PC9 cells treated with 5μM erlotinib exhibited significantly higher viability at day 3 compared to vector control cells under the same treatment (*P* < 0.05) (**Supplementary Figure S3B**). These findings collectively suggest that *TNNC1* enhances LUAD cell resistance to erlotinib.

Tumor-associated macrophage M2 polarization is implicated in tumor progression and chemotherapy resistance (41). This study assessed whether *TNNC1* affects LUAD biological functions through M2 tumor-associated macrophage (TAM) polarization. xCell analysis showed that patients with high *TNNC1* expression experienced more pronounced immune cell infiltration than those with low expression (**Figure 9L**), including significant enrichment of total macrophages and M2 polarization (**Figure 9M**). Immune-related genes, such as immune stimulatory and inhibitory genes, chemokines, and human leukocyte antigens were generally upregulated in the high *TNNC1* expression group (**Figure 9N**). These data suggest a strong association between *TNNC1* and M2 TAM polarization. The relationship between *TNNC1* expression in M2 cells and LUAD patient survival was explored. Using the TIMER2.0 database to integrate M2 cell infiltration levels and *TNNC1* transcriptional expression, LUAD patients were categorized into four groups. Results indicated that patients with a *TNNC1*+ & M2+ status had significantly poorer survival outcomes (**Figures 9O, P**). It was hypothesized that *TNNC1* could influence macrophage M2 polarization. To evaluate this, THP-1 monocytes were treated with IL-3 to induce M2 polarization, then transfected with *TNNC1*-specific siRNA. IL-3 treatment raised levels of M2 markers, including CD206, Ym1, and Arg1, while *TNNC1* knockdown reversed these increases (**Figure 9Q**). To directly confirm the role of *TNNC1* in promoting macrophage M2 polarization, we overexpressed *TNNC1* in THP1 cells and assessed its effects on M2 phenotype markers. Western blot confirmed successful expression of HA-tagged *TNNC1* in THP1 cells (**Supplementary Figure S1C**). Flow cytometry analysis demonstrated that *TNNC1* overexpression significantly increased the percentage of CD163+/CD206+ THP1 cells under IL-4/IL-13 stimulation compared to control cells (*P* < 0.01) (**Supplementary Figure S1C**). qPCR analysis further revealed that *TNNC1* overexpression significantly upregulated mRNA expression of M2-related markers including Arg1 (*P* < 0.001), IL-10, TGF-β, CCL17, and CCL22 (all *P* < 0.05) (**Supplementary Figure S1D**). Taken together, these findings provide direct evidence that *TNNC1* positively regulates macrophage M2 polarization.

In conclusion, *TNNC1* may contribute to erlotinib resistance in LUAD cells by promoting macrophage M2 polarization.

## 4 Discussion

LUAD is the predominant subtype of NSCLC, its marked heterogeneity, high recurrence rate, and poor prognosis present significant clinical challenges (1, 2). Accurately predicting the prognosis of patients with LUAD is essential for developing personalized treatment strategies. With advances in gene expression profiling and bioinformatics, large-scale data analyses focused on specific genetic features have provided a robust molecular foundation for prognosis prediction, guiding treatment decisions, and improving patient survival (3).

Our study focuses on *TNNC1* as a key tumor suppressor gene in LUAD. We confirmed through machine learning residual analysis that *TNNC1* is significantly downregulated in LUAD tissues, consistent with its function as a tumor suppressor gene. Notably, our findings are not the first to identify *TNNC1*’s prognostic value, as previous studies have already explored its prognostic significance in the same dataset. Lu et al. (2020) initially demonstrated significant expression differences of *TNNC1* between paired normal lung tissues and LUAD tissues, and that downregulation of *TNNC1* was closely correlated with increased mortality in LUAD patients (42). They demonstrated that there is a mutual inhibitory relationship between *TNNC1* expression and the KRAS signaling pathway, where KRAS suppression leads to enhanced *TNNC1* expression, while *TNNC1* overexpression in turn inhibits KRAS G12D-mediated anchorage-independent growth of NIH3T3 cells (42).

Our research significantly expands the understanding of *TNNC1*’s role in LUAD, particularly by revealing the association between *TNNC1* and EGFR-TKI resistance and tumor-associated macrophage polarization, areas not explored in previous studies. We observed that *TNNC1* is highly expressed in erlotinib resistance datasets, a finding consistent with the pattern proposed in previous studies where genes associated with drug resistance often exhibit low expression in cancer tissues but high expression in resistant cells (15, 40). Regarding the molecular mechanism of *TNNC1* in EGFR-TKI resistance, our data support the following explanation: *TNNC1* may participate in the development of resistance by regulating autophagy processes. Studies have shown that *TNNC1* protects non-small cell lung cancer cells from apoptosis by promoting gemcitabine-induced autophagy (43). A similar mechanism may exist in EGFR-TKI resistance, where upregulated *TNNC1* may promote cancer cell survival by enhancing autophagic flux, thereby conferring resistance to EGFR-TKIs such as erlotinib. The mechanisms of acquired resistance to EGFR-TKIs are complex and diverse, including T790M secondary mutations, MET amplification, and bypass signaling activation (15). *TNNC1* may participate in regulating these bypass signaling pathways, such as PI3K/AKT or MAPK pathways, thereby affecting the efficacy of EGFR-TKIs (44).

More importantly, our study is the first to reveal the close connection between *TNNC1* and M2 polarization of TAMs. TAMs predominate in the tumor microenvironment, with M2-type TAMs typically exhibiting pro-tumor characteristics, participating in promoting angiogenesis, immune suppression, and tumor metastasis (28, 41). Our data show that in the LUAD microenvironment, *TNNC1* may participate in regulating the process of macrophage polarization toward the M2 phenotype. The combined presence of *TNNC1*+ and M2+ exacerbates the adverse impact on LUAD patient survival, suggesting that *TNNC1* not only affects disease progression through direct action on tumor cells but also exerts indirect influence by regulating the tumor immune microenvironment. At the molecular level, *TNNC1* may participate in macrophage polarization through the following mechanisms: First, as a calcium metabolism-related protein, *TNNC1* may regulate calcium signaling in the tumor microenvironment, thereby affecting macrophage differentiation and polarization (4, 45). Calcium ions are important second messengers involved in regulating various cellular processes, including macrophage function and phenotypic transformation (45, 46). Second, *TNNC1* may regulate macrophage polarization by influencing cytokine networks. Studies have shown that in the tumor microenvironment, M2 macrophage polarization is regulated by various cytokines such as IL-4, IL-13, and the STAT6/C/EBPβ signaling pathways (29). *TNNC1* may participate in the regulation of these signaling pathways, thereby promoting M2 polarization. Additionally, *TNNC1* may indirectly regulate macrophage polarization by influencing interactions between tumor cells and macrophages, for example, by regulating the cytokine spectrum secreted by tumor cells or by affecting the expression of cell surface molecules (41). Based on our findings, targeting *TNNC1* may provide a novel strategy to overcome erlotinib resistance and inhibit macrophage polarization in LUAD patients, thereby improving patient survival rates. Potential intervention approaches include: developing specific inhibitors targeting *TNNC1*; combined use of EGFR-TKIs and autophagy inhibitors, such as chloroquine or hydroxychloroquine, to overcome *TNNC1*-mediated autophagy-related resistance (43).

Other genes identified in our nine-gene calcium-related prognostic signature also have important biological functions. S100 family members (*S100P* and *S100A10*) regulate key cellular processes such as cell cycle progression and differentiation (47). *S100P*, significantly upregulated in early-stage LUAD, promotes cancer progression by activating the PI3K/AKT pathway (44, 48) and interacts with interferon β (IFN-beta), indicating its potential role in lung cancer progression (49). *S100A10* (p11) plays a pivotal role in tumor invasion and metastasis by binding to annexin A2 (50), promoting extracellular matrix degradation and metastasis (51). *SLC34A2*, a pH-sensitive sodium-dependent phosphate transporter (52), is associated with lung cancer (53), and the *SLC34A2*-ROS1 fusion gene can induce crizotinib resistance and enhance carcinogenicity (54, 55). *CAMK2N1* influences cell cycle progression and epithelial-mesenchymal transition (56) through the MEK/ERK and Notch-1 pathways (57). *PKP2* accelerates tumor progression by promoting EGFR phosphorylation and activation (58, 59). *CFTR* gene methylation occurs in various tumors, including lung cancer, serving as a mechanism by which tumor cells suppress tumor suppressor genes (60–63). *MAPK10* plays an important role in biochemical signal integration (64, 65), while *CDKN3* is overexpressed in multiple cancers and correlates with poor prognosis (66–68).

Despite our significant progress, there are several limitations to our study. (1) potential selection biases despite our rigorous screening criteria and batch effect correction; (2) limitations of the CRPGscore predictive model, which shows good but not perfect performance that could benefit from validation in larger cohorts; (3) the need for further *TNNC1* functional characterization, particularly through *in vivo* models to better understand its impact on the tumor microenvironment; and (4) the requirement for additional preclinical and clinical validation of the five potential therapeutic compounds identified through CMap analysis.

In conclusion, our study indicated the complex function of *TNNC1* as a key tumor suppressor gene in LUAD, not only participating in the regulation of tumor cell proliferation and apoptosis but also in tumor microenvironment shaping and drug resistance development. Targeting *TNNC1* and its related pathways may provide new therapeutic strategies for LUAD patients, especially those who have developed resistance to existing treatments.

## Data Availability

The original contributions presented in the study are included in the article/**Supplementary Material**. Further inquiries can be directed to the corresponding authors.

## Supplementary material

Supplementary Figure 1 Gene set enrichment analysis of high and low risk groups. **(A)** GSVA analysis. **(B)** KEGG analysis, Red indicates that the pathway is activated in the high-risk group (as a result of up-regulated gene enrichment), blue indicates that it is inhibited in the high-risk group (as a result of up-regulated gene enrichment), and green indicates that the pathway has both up-regulated and down-regulated genes.

Supplementary Figure 2 Analysis of druggable genes in LUAD by MR and PheWAS. **(A)** Forest plot for MR results between cis-eQTL brain and LUAD. **(B)** Binary traits and continuous traits PheWAS association with *TNNC1*.

Supplementary Figure 3
*TNNC1* overexpression promotes M2 Macrophage polarization and enhances drug sensitivity in PC9 cells. **(A)** Western blot analysis of *TNNC1*-HA protein expression in PC9 and THP1 cell lines. The overexpression (OE) groups show clear *TNNC1*-HA protein expression detected by anti-HA antibody at approximately 25kDa in lanes 2 and 3 with increasing loading amounts, while the control (CT) groups show no detectable *TNNC1*-HA expression. Anti-GAPDH (35kDa) was used as a loading control for both cell lines, confirming the presence of protein in all samples. Three different loading amounts (1, 2*, 3*) were used for both the overexpression and control conditions. **(B)** Cell viability assay (CCK-8) showing the effect of *TNNC1* overexpression on PC9 cell growth with and without treatment. The graph displays cell viability (OD 450nm) over 3 days for four conditions: PC9-Vector with 0μM treatment (black circles), PC9-Vector with 5μM treatment (green squares), PC9-*TNNC1*-OE with 0μM treatment (red triangles), and PC9-*TNNC1*-OE with 5μM treatment (blue inverted triangles). **(C)** Flow cytometry analysis showing the differentiation of THP1 cells under various conditions. Left panels display representative flow cytometry dot plots of CD206 and CD163 expression in THP1 cells treated with: IL-4/IL-13 alone (top row), IL-4/IL-13 with *TNNC1*-CT (control, middle row), and IL-4/IL-13 with TNNC1-OE (overexpression, bottom row). Each condition shows three technical replicates with the percentage of CD206+/CD163+ cells indicated in the upper right quadrant. Right panel shows the quantification of CD206+/CD163+ THP1 cells across the three treatment groups. **(D)** qPCR analysis of M2 macrophage marker expression in differentiated THP1 cells. The graph shows relative mRNA expression levels of Arg1, IL-10, TGF-β, CCL17, and CCL22 in Control (black), Vector (pink), and *TNNC1*-OE (blue) groups. Statistical analyses were performed using Student’s t-test to compare differences between groups. **P* < 0.05, ***P* < 0.01, and ****P* < 0.001.
