# Supplementary figures and images for: Prognostic significance of calcium-related genes in lung adenocarcinoma and the role of TNNC1 in macrophage polarization and erlotinib resistance
Source: Front Immunol. 2025 May 13;16:1509222. doi: 10.3389/fimmu.2025.1509222 (PMC12106437; doi:10.3389/fimmu.2025.1509222)

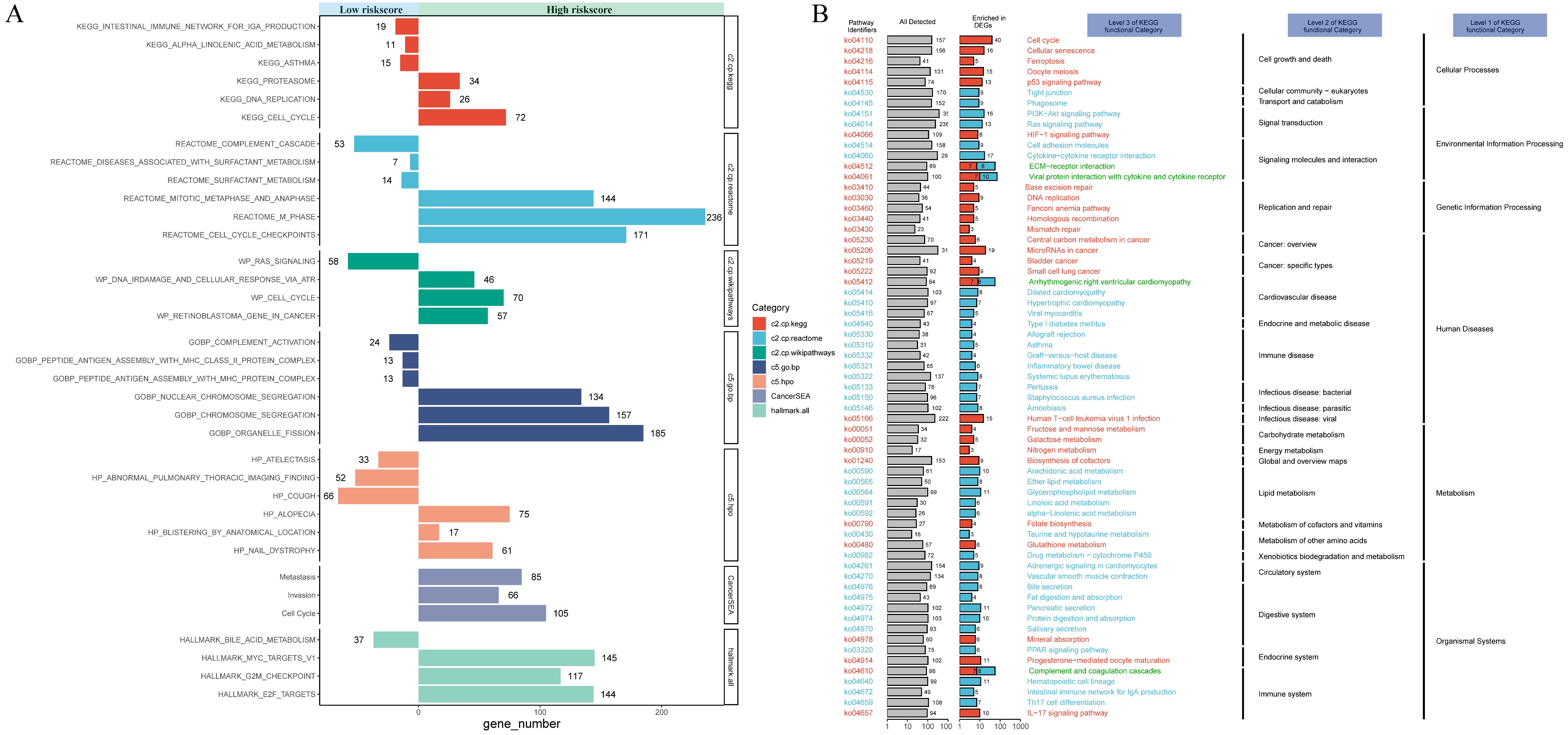

Supplement: Supplementary Figure 1 — Gene set enrichment analysis of high and low risk groups. (A) GSVA analysis. (B) KEGG analysis, Red indicates that the pathway is activated in the high-risk group (as a result of up-regulated gene enrichment), blue indicates that it is inhibited in the high-risk group (as a result of up-regulated gene enrichment), and green indicates that the pathway has both up-regulated and down-regulated genes. [file Image1.jpeg]

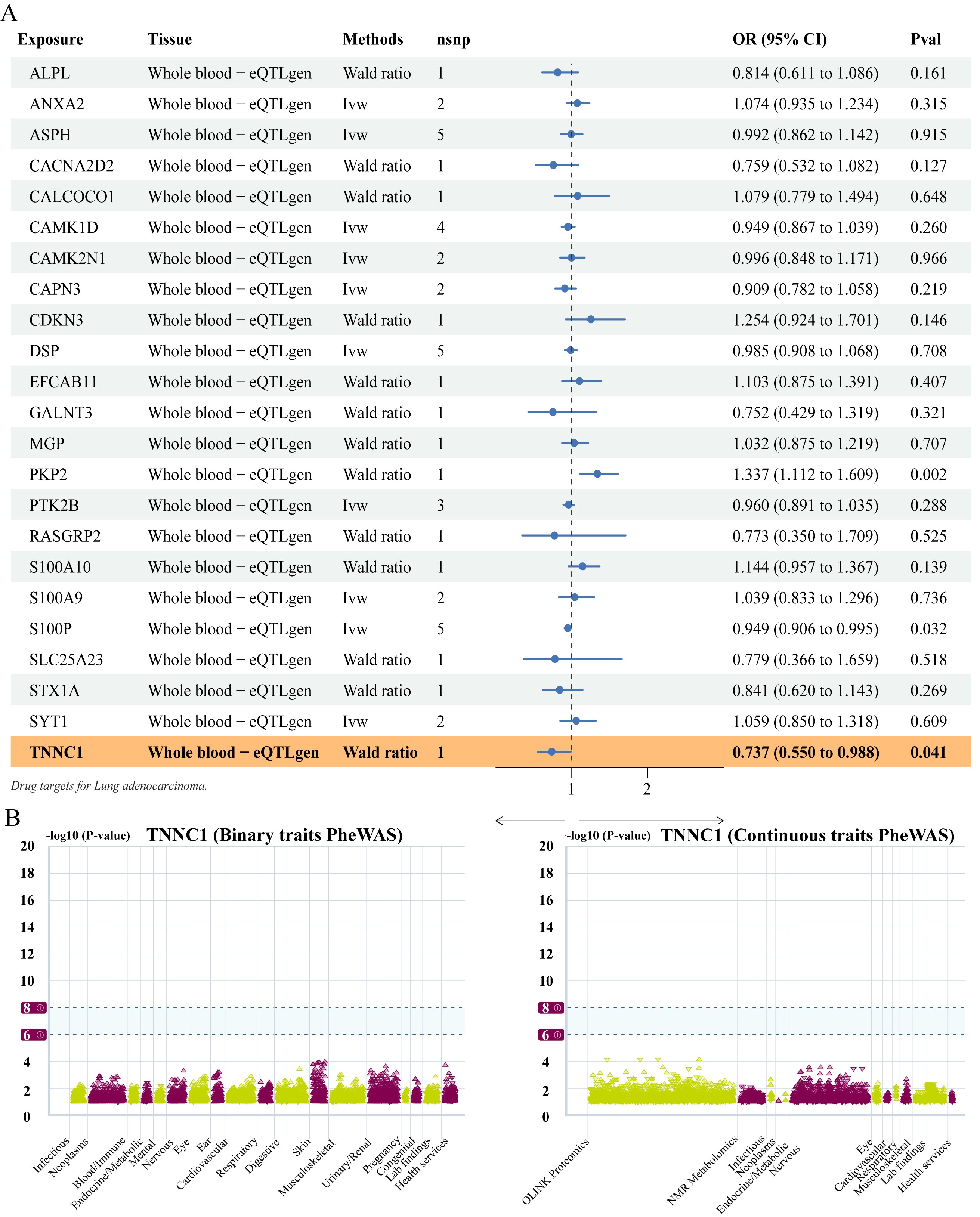

Supplement: Supplementary Figure 2 — Analysis of druggable genes in LUAD by MR and PheWAS. (A) Forest plot for MR results between cis-eQTL brain and LUAD. (B) Binary traits and continuous traits PheWAS association with TNNC1. [file Image2.jpeg]

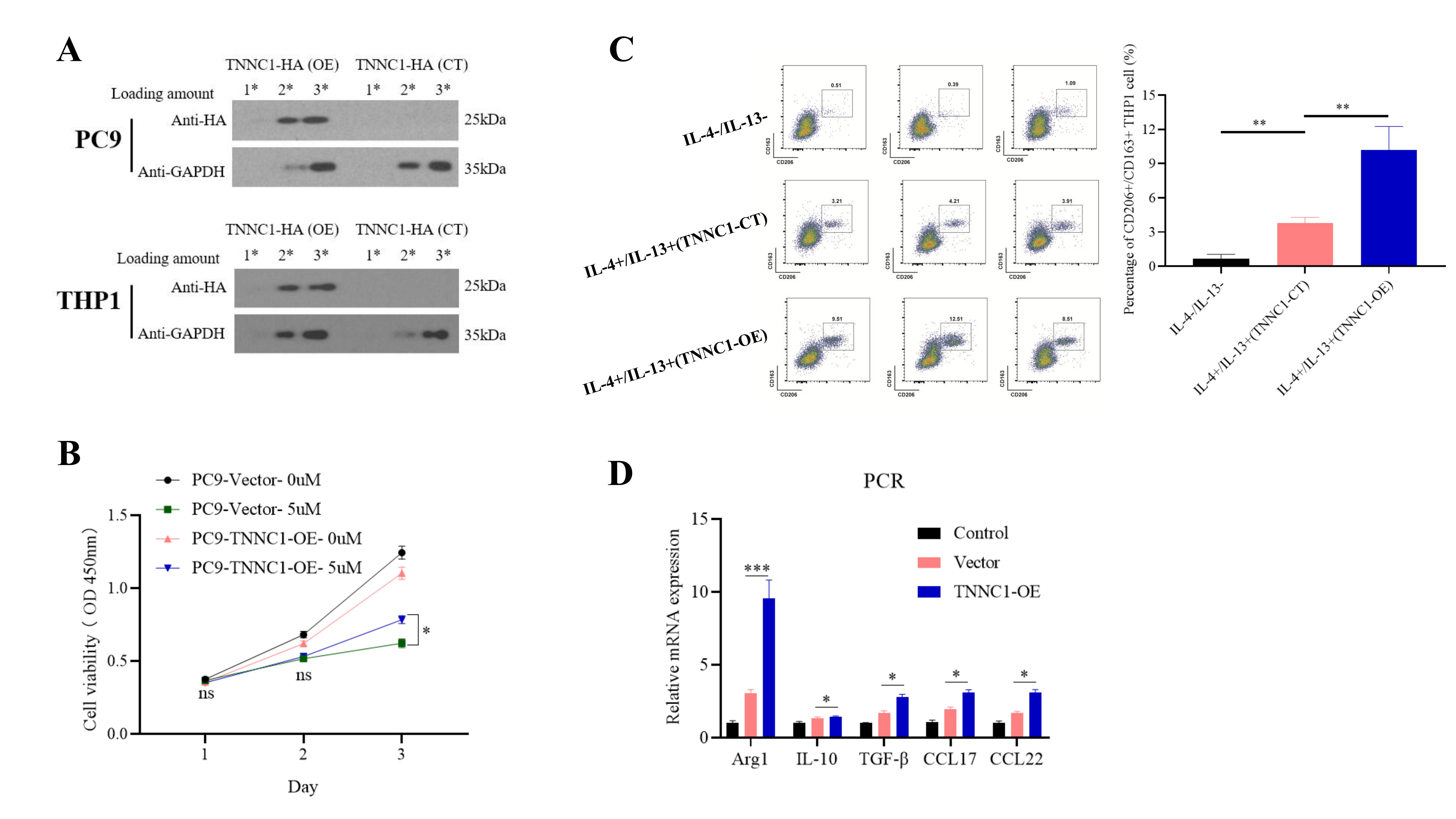

Supplement: Supplementary Figure 3 — TNNC1 overexpression promotes M2 Macrophage polarization and enhances drug sensitivity in PC9 cells. (A) Western blot analysis of TNNC1-HA protein expression in PC9 and THP1 cell lines. The overexpression (OE) groups show clear TNNC1-HA protein expression detected by anti-HA antibody at approximately 25kDa in lanes 2 and 3 with increasing loading amounts, while the control (CT) groups show no detectable TNNC1-HA expression. Anti-GAPDH (35kDa) was used as a loading control for both cell lines, confirming the presence of protein in all samples. Three different loading amounts (1, 2*, 3*) were used for both the overexpression and control conditions. (B) Cell viability assay (CCK-8) showing the effect of TNNC1 overexpression on PC9 cell growth with and without treatment. The graph displays cell viability (OD 450nm) over 3 days for four conditions: PC9-Vector with 0μM treatment (black circles), PC9-Vector with 5μM treatment (green squares), PC9-TNNC1-OE with 0μM treatment (red triangles), and PC9-TNNC1-OE with 5μM treatment (blue inverted triangles). (C) Flow cytometry analysis showing the differentiation of THP1 cells under various conditions. Left panels display representative flow cytometry dot plots of CD206 and CD163 expression in THP1 cells treated with: IL-4/IL-13 alone (top row), IL-4/IL-13 with TNNC1-CT (control, middle row), and IL-4/IL-13 with TNNC1-OE (overexpression, bottom row). Each condition shows three technical replicates with the percentage of CD206+/CD163+ cells indicated in the upper right quadrant. Right panel shows the quantification of CD206+/CD163+ THP1 cells across the three treatment groups. (D) qPCR analysis of M2 macrophage marker expression in differentiated THP1 cells. The graph shows relative mRNA expression levels of Arg1, IL-10, TGF-β, CCL17, and CCL22 in Control (black), Vector (pink), and TNNC1-OE (blue) groups. Statistical analyses were performed using Student’s t-test to compare differences between groups. *P < 0.05, **P < 0.01, and [file Image3.jpeg]
